# Novel Approach to Maintaining Patency of an Abdominal Aortic Graft Required for Emergent Cannulation

**DOI:** 10.1016/j.atssr.2023.08.004

**Published:** 2023-09-19

**Authors:** Blaine Johnson, David Onsager, Avery Tung, Rolla Zarifa, Luis Landeras, Sean Pinney, Ross Milner, Valluvan Jeevanandam

**Affiliations:** 1 Perfusion Services, UChicago Medicine, Chicago, Illinois; 2 Department of Surgery, University of Chicago, Chicago, Illinois; 3 Department of Anesthesia and Critical Care, University of Chicago, Chicago, Illinois; 4 Department of Radiology, University of Chicago, Chicago, Illinois; 5 Department of Medicine, University of Chicago, Chicago, Illinois

## Abstract

A 32-year-old woman with end-stage heart failure and complex anatomy underwent placement of a vascular graft to facilitate arterial cannulation during planned heart transplantation. The procedure involved anastomosis of a prosthetic graft to the abdominal aorta. The vascular graft was plugged with a silicone Foley catheter containing a stopper to maintain patency. This novel approach was safe when performed by a multidisciplinary surgical team experienced in vascular and adult congenital cardiac surgical procedures.

With advances in diagnosis and management, many children with congenital heart defects reach adulthood.^1^ Patients with adult congenital heart disease (ACHD) require multiple palliative or corrective cardiac surgical procedures if end-stage heart failure develops. In patients with single-ventricle physiology palliated by Fontan-type operations, heart transplantation is an effective treatment, but successful management requires multidisciplinary collaboration. We describe a patient with Ebstein anomaly who presented with challenging access required for cardiopulmonary bypass (CPB) or extracorporeal membrane oxygenation (ECMO) arterial cannulation.

A 32-year-old woman with a history of Ebstein anomaly and pulmonary atresia presented for heart transplantation. She had undergone several previous palliative procedures, including right-sided Blalock-Taussig shunt, right ventricular outflow tract patch for pulmonary atresia, and anastomosis between the superior vena cava/right atrium/right pulmonary artery. She then underwent a bidirectional Glenn procedure, followed by a complete lateral tunnel Fontan. Severe biventricular dysfunction subsequently developed, and she was listed for heart transplantation.

This patient posed several anatomic challenges to successful heart transplantation (Figure 1). Because of a right innominate artery pseudoaneurysm, her aorta and innominate vein were adherent to the sternum. In addition, her right innominate artery had been previously divided, and her left axillary artery and both iliac arteries were <5 mm in diameter. Peripheral cannulation in case of catastrophic bleeding during redo sternotomy was thus impossible. To facilitate heart transplantation, we thus performed a preliminary procedure to create a conduit for emergency CPB. This procedure was accomplished by anastomosis of a plugged prosthetic graft to the aorta and positioning of the plugged end in the left femoral area for emergent access if needed (Figure 2).

**Figure 1:**
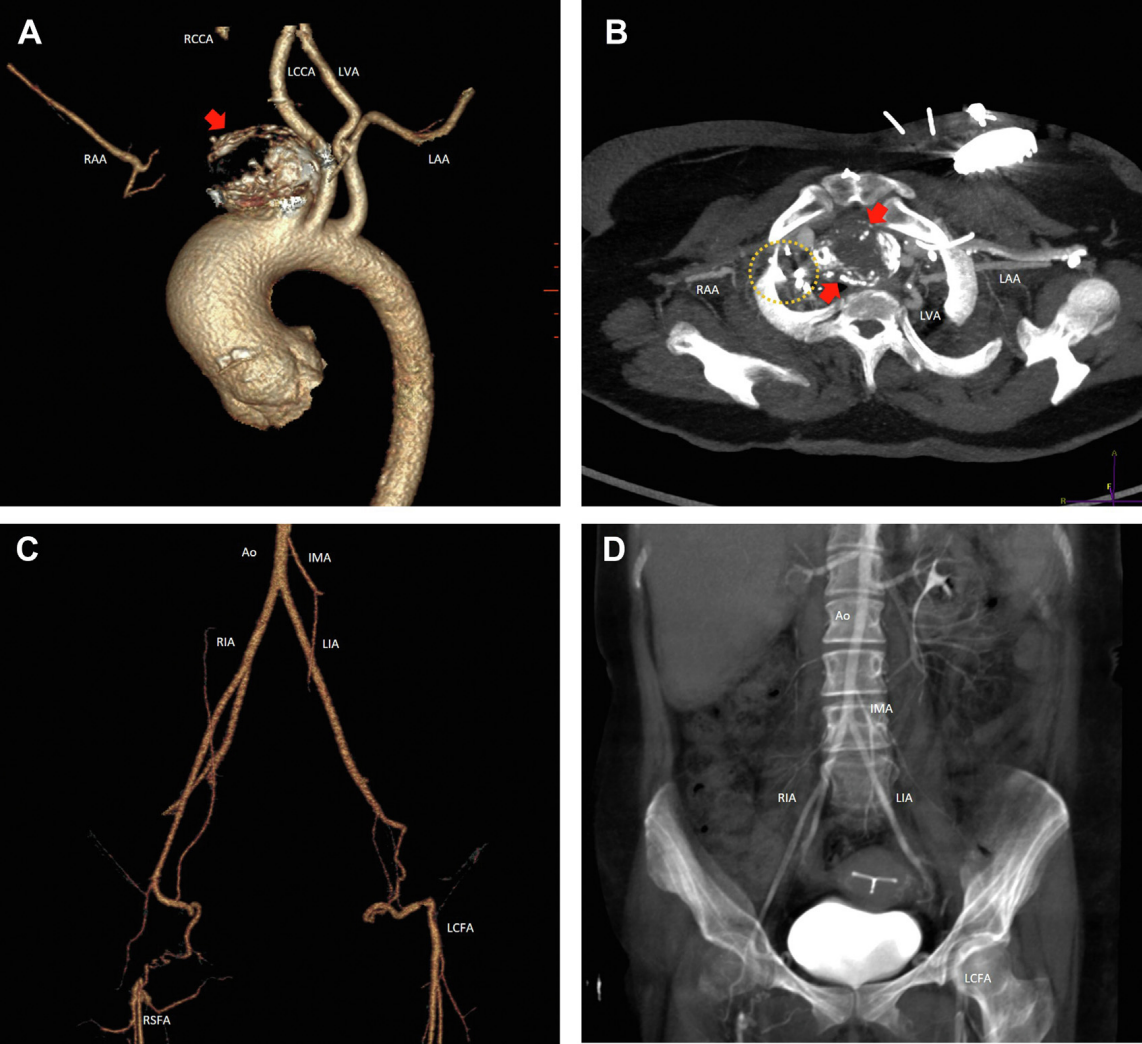
Preoperative imaging. (A) Three-dimensional volume rendering and (B) axial oblique maximal intensity projection images demonstrate a thrombosed and calcified right innominate artery aneurysm (arrows) noncontinuous with the right subclavian with multiple metallic clips in place (dashed circle). (C) Three-dimensional volume rendering and (D) average coronal oblique slab images reveal a diminutive distal abdominal aorta and small-caliber right and left iliac arteries. (Ao, aorta; IMA, inferior mesenteric artery; LAA, left axillary artery; LCCA, left common carotid artery; LCFA, left common femoral artery; LIA, left iliac artery; LVA, left vertebral artery; RAA, right axillary artery; RCCA, right common carotid artery; RIA, right iliac artery; RSFA, right superficial femoral artery.)

**Figure 2:**
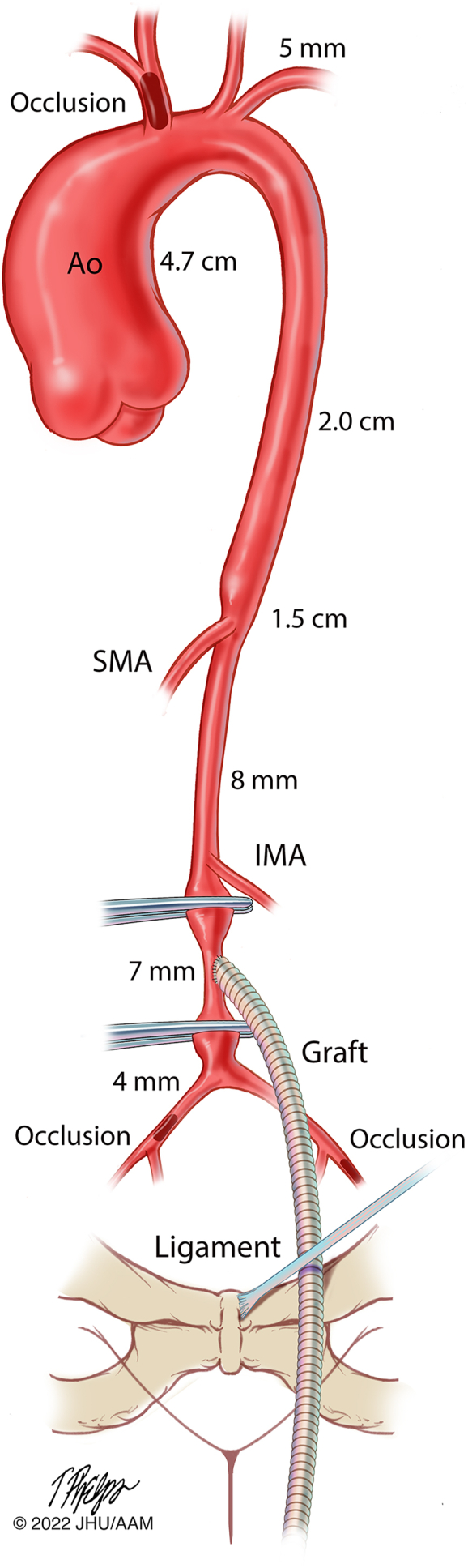
Complex cardiac and vascular anatomy with abdominal aortic vascular access graft. A vascular graft is sutured to the abdominal aorta (Ao) and tunneled into the proximal thigh. The diminutive vasculature is unreliable for emergent arterial cannulation. (IMA, inferior mesenteric artery; SMA, superior mesenteric artery.) Illustration: Tim Phelps © 2022 JHU AAM, Department of Art as Applied to Medicine, The Johns Hopkins University School of Medicine.

A midline incision was made from just above the umbilicus to the symphysis pubis. The aorta and proximal common iliac arteries were exposed through a transverse incision, and a tunnel was created in the retroperitoneum from the aorta to the left femoral area. After heparinization, the aorta was clamped proximally below the level of the inferior mesenteric artery and distally at the level of the aortic bifurcation. An 8 mm Dacron graft was then anastomosed to the aorta in an end-to-side fashion. The free distal end was passed through the retroperitoneal tunnel and positioned in the left groin posterior to the inguinal ligament. A pocket was created superficial to the muscular fascia.

To occlude the free end of the graft and to preserve patency, 3 mm of silicone adhesive (Medtronic) was used to fill a 24F silicone Foley catheter (Medline Industries). A 10F Shiley intubating stylet (Covidien) was then passed through the Foley catheter to serve as a stylet. The distal tip of the catheter was then inserted into the open end of the graft and advanced to a position approximately 1 mm distal to the junction between the graft and the aorta. The intubating stylet was removed, and the catheter was secured within the graft with silk ties. The terminal end of the graft was positioned within the pocket, and the fascia layer was closed over the graft (Figure 3).

**Figure 3:**
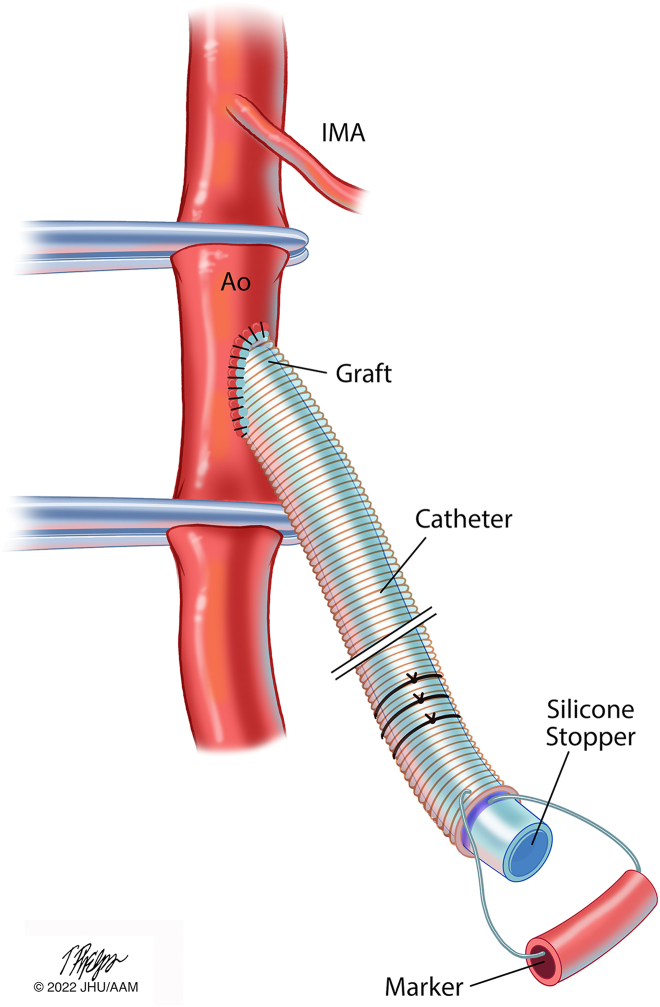
Vascular access graft residing in proximal thigh. A silicone Foley catheter containing a silicone stopper is secured within the vascular graft to maintain the patency of the graft lumen. A stainless steel suture is attached with a portion of a red rubber catheter to aid in locating the graft. (Ao, aorta; IMA, inferior mesenteric artery.) Illustration: Tim Phelps © 2022 JHU AAM, Department of Art as Applied to Medicine, The Johns Hopkins University School of Medicine.

Approximately 48 hours later, the patient underwent successful heart transplantation. A fourth time redo median sternotomy was performed with an oscillating saw. CPB was established routinely through central arterial and bicaval cannulation with no complications.

The graft was removed after implantation of the donor heart and when the patient was hemodynamically stable. The skin incision was opened, the free end of the graft was exposed, and the silicone Foley catheter was removed from the graft lumen. After removal, backbleeding exhibited excellent distal flow, and the graft was tied off (Video). The patient was discharged in good condition on postoperative day 17 with no complications from the graft procedure.

## Comment

This case report highlights several challenges in managing patients with ACHD with anatomic and vascular anomalies who have undergone prior palliative procedures. In this case, the patient had been turned down at several large transplant centers with extensive ACHD experience.

In patients who have undergone prior palliative procedures, the preoperative assessment for transplantation should include the adequacy of peripheral venous and arterial sites for CPB, ECMO, or both if emergent cannulation is needed.^2^ In our patient, the lack of vascular access to support CPB or ECMO prevented the patient from being listed at several centers. The patient's DuBois body surface area was calculated to be 1.66 m^2^, which would result in a calculated flow of 3.99 L/min at a 2.4 cardiac index.

We addressed the lack of access by creating an aortic conduit during a novel preparatory procedure. The timing of this novel intervention was difficult as the optimal timing for transplantation in patients with complex ACHD is unknown and it is infrequently performed.^3^ This preparatory step allowed the patient to be upgraded from United Network for Organ Sharing status 4 to a status 2 exception. Although unknown at the time of the intervention, an organ offer with successful transplantation would coincidentally follow 48 hours later.

We have previously described using an arterial conduit to allow the insertion of a minimally invasive, ambulatory, counterpulsation heart assist system.^4^ In this approach, a hemostatic plug fits snugly within the graft, occupying nearly the entire volume and surrounding the pump driveline. Thrombosis of the graft is unlikely, and the conduit can be used for other circulatory support devices. In our first-in-human experience with this counterpulsation device, escalation of care 28 days after implantation was achieved with an Impella 5.0 (Abiomed).^4^ This experience suggested that occlusion of a vascular graft with an intragraft plug would result in a functioning vascular access graft for arterial access after a similar period.

The primary limitation of this approach is the risk involved with anesthesia and the operation for aortic conduit creation. To that end, our patient was optimized preoperatively by the heart failure service and managed intraoperatively by a cardiac anesthesia team. Unfortunately, although we prepared to use this novel graft, it was ultimately not required. Therefore, we cannot comment on how well it provided support without complication.

This case also highlights the importance of a multidisciplinary approach to complex cardiac and vascular care. Coordination between cardiac surgery, vascular surgery, cardiac anesthesia, and perfusion services within our heart and vascular center allowed a novel approach to facilitate heart transplantation in a patient previously deemed too high risk. In this setting, placing an abdominal vascular graft for emergent cannulation may be an option for other patients presenting for transplantation after congenital repair.

We describe using an abdominal aortic graft to allow emergent arterial access and to permit high-risk dissection for transplantation after congenital cardiac abnormalities and multiple palliative procedures. This approach may reduce the risk of complex transplantation in patients with congenital heart disease and allow more patients access to potentially lifesaving transplants. In addition, when it is performed by a dedicated, multidisciplinary surgical team experienced in vascular and adult congenital cardiac surgical procedures, this approach can be used for cannulation access in other complex patients.
